# Effects of physical activity and feed and water restriction at reimplanting time on feed intake patterns, growth performance, and carcass characteristics of finishing beef steers

**DOI:** 10.1093/tas/txac008

**Published:** 2022-01-16

**Authors:** Cory L Helmuth, Dale R Woerner, Michael A Ballou, Jeff L Manahan, Carley M Coppin, Nathan S Long, Ashley A Hoffman, James Daniel Young, Taylor M Smock, Kristin E Hales

**Affiliations:** 1 Department of Animal and Food Sciences, Texas Tech University, Lubbock, TX 79409, USA; 2 Department of Veterinary Sciences, Texas Tech University, Lubbock, TX 79409, USA

**Keywords:** dry matter intake, feedlot steers, growth, reimplant

## Abstract

In the feedlot, there can be a decrease in dry matter intake (DMI) associated with reimplanting cattle that negatively affects growth performance. This study was conducted to determine the mechanisms causing a decrease in DMI after reimplanting and identify a strategy to mitigate the decrease. Crossbred steers (*n* = 200; 10 pens/treatment; initial bodyweight [BW] = 386 ± 4.9 kg) were used in a randomized complete block design experiment. Cattle were implanted with Revalor-IS on day 0. Treatments included a Revalor-200 implant on day 90 before feeding with the following management practices imposed: 1) steers were returned to their home pen immediately after reimplant (PCON); 2) steers were placed in pens and restricted from feed and water for 4 h (RES); 3) steers were walked an additional 805 m after reimplant and then returned home (LOC); 4) steers were restricted from feed and water for 4 h and walked an additional 805 m (RES + LOC); 5) steers were given an oral bolus of *Megasphaera elsdenii* (Lactipro; MS Biotec, Wamego, KS) and were restricted from feed and water for 4 h, and then walked an additional 805 m (LACT). One hundred steers were given an ear tag to record minutes of activity (ESense Flex Tags, Allflex Livestock Intelligence, Madison, WI). As a percentage of BW, DMI was 5% greater (*P* = 0.01) from reimplant to end for PCON vs. RES, LOC, and RES + LOC treatments. Likewise, as a percentage of BW, DMI was 6.6% greater (*P* = 0.03) from reimplant to end and 4.0% greater (*P *= 0.05) overall for the PCON treatment vs. the LOC treatment. Overall, DMI as a percentage of BW was 3.3% greater (*P* = 0.02) for PCON vs. RES, LOC, and RES + LOC treatments. There was an increase in G:F from reimplant to end (*P *= 0.05) for RES + LOC vs. the LACT treatment. From these data, we conclude that restricting cattle from feed and water for 4 h after reimplanting did not alter subsequent DMI. Increasing locomotion had the greatest negative effect on DMI and growth performance. Management strategies to decrease locomotion associated with reimplanting would be beneficial to DMI and overall growth performance of finishing beef steers.

## INTRODUCTION

Use of implants in finishing cattle has been approved by the U.S. Food and Drug Administration since 1956 (Preston, 1999). Approximately 90% of feedlot cattle are given a single anabolic implant and 79% of steers and 98% of heifers that weigh less than 318 kg are given at least 2 implants (APHIS, 2013). Implants increase average daily gain (ADG), gain:feed (G:F), and improve yield grade (YG) compared to nonimplanted cattle (Smith et al., 2018). Likewise, the use of multiple implants increases final shrunk body weight (BW), ADG, and G:F vs. using a single implant (Reinhardt and Wagner, 2014). Bartle et al. (1992) reported that trenbolone acetate (TBA) and estradiol-17β (E_2_) containing implants increased ADG and G:F by 15% to 20% in finishing cattle. Nonetheless, Wallace et al. (2008) noted that cattle consumed 0.2 kg less daily dry matter (DM) for 10 d following a reimplant. Llonch et al. (2018) reported that increased physical activity and locomotion were associated with decreased G:F. Another possible cause of decreased DMI associated with reimplanting is restricted access to feed and water during the reimplant process, thus causing cattle to rapidly consume feed once returned to their pen. The rapid consumption of a high-concentrate diet causes decreased ruminal pH, and increased production of lactic acid which may lead to ruminal acidosis, causing a subsequent decrease in DMI (Owens et al., 1998).

*Megasphaera elsdenii* is an important lactate-utilizing bacteria and can be beneficial during times of acidosis (Mobiglia et al., 2016). Henning et al. (2010) reported a decrease in lactic acid concentration in cattle, given *M. elsdenii* compared to cattle that did not consume it. Steers consuming a high-concentrate diet treated with *M. elsdenii* had a 21% increase in DMI and a subsequent increase in ADG compared to steers that did not consume the *M. elsdenii* containing DFM (Henning et al., 2010). Therefore, *M. elsdenii* has the potential to mitigate decreases in DMI associated with reimplanting finishing cattle. The objectives of this study were to identify the mechanisms that decrease DMI after reimplanting, identify a mitigation strategy to prevent the decrease, and increase overall growth performance of finishing beef steers.

## MATERIALS AND METHODS

Experimental procedures were approved by the Texas Tech University Institutional Animal Care and Use Committee (approval number 20031-03). The experiment was conducted at the Texas Tech University Burnett Center from September 2020 to May 2021.

### Animal Processing and Experimental Diets

Two hundred crossbred steers sourced from Southern Oklahoma (predominantly *Bos taurus*) were used for the study. Cattle arrived on September 11, 2020 (BW = 299 ± 39.4 SD). After arrival, steers were housed in soil-surface pens, provided ad libitum access to water, long-stem grass hay, and a 65% concentrate receiving diet that was fed at 1% of BW on the day of arrival. Within 24 h after arrival, steers were processed, given an identification tag, and individual BW was measured. Cattle were weighed using a hydraulic squeeze chute (Silencer, Moly Manufacturing, Lorraine, KS) with load cells that were calibrated before use with 454 kg of certified weight. All steers were vaccinated against infectious bovine rhinotracheitis virus, bovine viral diarrhea virus types I and II, bovine parainfluenza-3 virus, bovine respiratory syncytial virus (Vista 5 SQ; Merck Animal Health, Kenilworth, NJ), clostridial species (Vision 8 with Spur; Merck Animal Health), and *Mycoplasma bovis* (Myco-B ONE DOSE; American Animal Health, Inc. Grand Prairie, TX). Cattle also received fenbendazole (Safeguard; Merck Animal Health) and ivermectin (Vetrimec pour-on; Vet One, Boise, ID) for internal and external parasites. Ears of the cattle were palpated to check for any previous implants, and none were present.

### Experimental Design

Approximately 1 mo after arrival, an individual BW was collected and used to allocate steers to BW blocks. Three weeks before the start of the experiment, the steers were placed in partially slotted concrete pens and allowed to acclimate to their pen (*n* = 50 pens, 10 replications/treatment, 4 steers/pen) before the start of the experiment. The starting dates of the experiment were staggered for blocks 1 to 4, 5 to 7, and 8 to 10; however, the reimplant was administered on day 90 for all blocks of steers. Blocks 1 to 4 were on feed for 166 d, blocks 5 to 7 were on feed for 188 d, and blocks 8 to 10 were on feed for 166 d.

Five treatments were used in a randomized complete block design with pen as the experimental unit. All cattle were implanted with Revalor-IS [80 mg of TBA and 16 mg of Estradiol-17β E_2_] on day 0 when individual BW was collected before feeding. One hundred steers (20 steers/treatment; 2 steers/pen) were affixed with an ear-tag to continuously record physical activity via a 3-axis accelerometer (ESense Flex Tags, Allflex Livestock Intelligence, Madison, WI). The 3-axis accelerometer tags have been validated previously for quantification of time spent ruminating and minutes of activity (Wolfger et al., 2015). The treatments consisted of the following: 1) Revalor-200 (200 mg of TBA and 20 mg of E_2_) on day 90 before feeding, then steers were returned to their home pen after reimplant (PCON); 2) Revalor-200 on day 90 before feeding, steers were then placed in holding pens and restricted from feed and water for 4 h (RES); 3) Revalor-200 on day 90 before feeding, steers were walked an additional 805 m after reimplant and then returned to their home pen (LOC); 4) Revalor-200 on day 90 before feeding, steers were walked an additional 805 m and placed in holding pens and restricted from feed and water for 4 h (RES** + **LOC); and 5) Revalor-200 on day 90 with an oral bolus of *Megasphaera elsdenii* administered with a balling gun (1.0 × 10^10^ CFU/bolus, Lactipro; MS Biotec, Wamego, KS) before feeding, steers were walked an additional 805 m and placed in holding pens and restricted from feed and water for 4 h (LACT). Boluses were administered orally via bolus gun. One steer in the LACT treatment within blocks 7, 8, or 9 regurgitated the bolus, but we were uncertain which steer the bolus belonged to, so no data were excluded from the analysis.

Blocks 1 to 4 were reimplanted on January 27, 2021, blocks 5 to 7 were reimplanted on February 24, 2021, and blocks 8 to10 were reimplanted on March 10, 2021. Feed delivery occurred after the steers were removed from their pen and feed was delivered before they were returned. The quantity fed on the day of reimplant was based on the previous day’s feed call. The PCON treatment walked an average of 362 m from the time they left their pen until they returned their home pen; we estimate each pen of steers was back in their home pen within 15 min (time not measured). Cattle that were walked an additional 805 m were walked on a combination of soil and concrete surface. Cattle that were restricted from feed and water for 4 h were held in 9.0 × 3.6 m holding pens. Ears of the cattle were palpated on day 42 after initial implant and day 118 after reimplanting to check condition of the implant site after the initial and terminal implant. No missing implants or abscesses around the implant site were noted.

### Feeding Management

Steers were transitioned from the 65% concentrate diet to the final diet using a 4-step process (65%, 75%, 85%, and 90% concentrate diets) over 35 d. By October 15, 2020, steers were consuming a 90% concentrate diet that was fed throughout the study (Table 1). The diet was formulated to meet nutrient requirements (NASEM, 2016) for growing and finishing beef cattle and included 30 g/ton of monensin sodium with each steer consuming 265 mg of monensin daily (Rumensin 90, Elanco Animal Health, Greenfield, IN).

**Table 1.** Ingredient and nutrient composition of the finishing diet used to determine the effects of physical activity and feed and water restriction at reimplanting time in finishing beef steers ^1^

Item	Finishing diet
Ingredient, %	
Steam-flaked corn	64.39
Sweet Bran	20.26
Alfalfa hay	7.66
Yellow grease	2.84
Limestone	1.66
Urea	0.66
Supplement^2^	2.53
Analyzed nutrient composition^3^	
Diet DM, %	76.9
Crude protein, %	14.0
Neutral detergent fiber, %	16.6
Acid detergent fiber, %	7.2
Ash, %	4.5
Fat, %	5.6
Ca, %	0.65
P, %	0.35
NEm^4^, Mcal/kg	2.20
NEg^4^, Mcal/kg	1.48

Dry matter basis, except diet DM%.

Supplement supplied 5.99% potassium chloride, 44.40% crude protein, 3.82% sodium, 8.34 mg/kg cobalt carbonate, 395.00 mg/kg copper sulfate, 408.00 mg/kg iron sulfate, 764 mg/kg manganous oxide, 2.92 mg/kg selenium, 2,490.00 mg/kg zinc sulfate, and 30.0 mg/kg monensin sodium (Rumensin 90; Elanco Animal Health, Greenfield, IN) on a DM basis.

Analysis performed by Servi-Tech Laboratories, Amarillo, TX. Actual diet formulation based on weekly DM determinations.

NE_m_ and NE_g_ reported as tabular values (NASEM, 2016).

Throughout the study, cattle had ad libitum access to feed and fresh water. The feed bunks were evaluated each morning at 0730 h. The bunk management approach was to achieve ad libitum intake with minimal orts (< 0.5 kg) in the bunk at the time of feeding. Steers were fed once daily at 0800 h. Diets were mixed in a paddle-type mixer and conveyed to a tractor pulled mixer (Rotomix 84–8 wagon mixer; Rotomix, Dodge City, KS; scale readability ± 0.45 kg). The diets were sampled 3 times each week throughout the study and composited by week. The weekly sample was divided and one half of the sample was used to determine DM in a forced-air oven at 100 °C for 24 h (The Grieve Corporation; Round Lake, IL). The DM was used to calculate total DMI for each week. The second subsample was used for the commercial chemical analyses of crude protein, ADF, NDF, fat, starch, ash, Ca, and P concentration. On days when BW was collected, reimplant was administered, or when feed spoilage occurred, orts were removed from bunks, weighed, and dried in a forced-air oven as described previously and used to compute DMI. Additionally, DMI 7 d before and after reimplanting was monitored; whereby, orts from each pen were weighed and then returned to the bunk daily before feeding.

Final BW was carcass-adjusted by dividing the hot carcass weight (HCW) by the overall average dressing percent (65.51%). Carcass-adjusted ADG was calculated by subtracting initial BW from the carcass-adjusted final BW, and then divided by total days on feed. The carcass-adjusted G:F was calculated by dividing the carcass-adjusted ADG by the DMI from day 0 to end.

### Statistical Analysis

Six steers were removed from the study (4 mortalities, 1 injury, and 1 for lameness) and the data (1 steer from PCON, 1 steer was from LOC, and 4 steers were from RES + LOC). On reimplant day, 1 pen in block 6 on the RES treatment was mistakenly walked an extra 240 m.

Live growth performance and carcass-adjusted data were analyzed as a randomized complete block design using PROC MIXED in SAS (SAS Inst. Cary, NC). Pen was the experimental unit for all analyses, and the model included the fixed effect of treatment and the random effect of BW block. Because of differences in ADG among treatments from day 0 to reimplant, it was included as a covariate for ADG from reimplant to end and overall ADG. Likewise, because of differences in G:F among treatments from day 0 to reimplant, it was included as a covariate for G:F from reimplant to end and overall G:F.

Least squares means for growth performance, activity, and carcass characteristics were evaluated using preplanned, single degree-of-freedom contrasts: 1 = PCON vs. the average of RES, LOC, and RES + LOC to evaluate whether there was a difference in DMI associated with varying practices on reimplant day; 2 = RES vs. LOC to evaluate whether restriction from feed and water or additional locomotion caused a difference in DMI; 3 = RES + LOC vs. LACT to evaluate whether administering an oral bolus of *M. elsdenii* was an effective mitigation strategy to prevent a decrease in DMI; 4 = PCON vs. RES to determine what percentage of the decrease of DMI is associated with restricting from feed and water; 5 = PCON vs. LOC to determine what percentage of the decrease of DMI is associated with locomotion.

Dry matter intake (kg and percentage of BW) data were analyzed using PROC MIXED with repeated measures 7 d before and 7 d after reimplanting. The model included treatment, day, and the interaction of treatment × day, with block included as a random effect. Pen within treatment was the subject of the repeated measure and several covariance structures were tested. An autoregressive covariance structure resulted in the smallest Akaike and Schwarz Bayesian criteria and was considered the most appropriate for analysis. The SLICE option of the MIXED procedure was used to separate treatment × day effects.

Activity data were analyzed using PROC MIXED with repeated measures 7 d before and 7 d after reimplanting. The model included treatment, day, and the interaction of treatment × day, with block included as a random effect. Pen within treatment was the subject of the repeated measure and several covariance structures were tested. An autoregressive covariance structure resulted in the smallest Akaike and Schwarz Bayesian criteria and was considered the most appropriate for analysis. The SLICE option of the MIXED procedure was used to separate treatment × day effects.

Data from 28 carcasses were unattainable from the commercial abattoir because of EID error (6 carcasses from the PCON, 5 from the RES, 4 from the LOC, 9 from the RES + LOC, and 4 from the LACT treatment). An entire pen of carcass data was lost from the RES + LOC treatment; otherwise, data were obtained from at least 2 steers/pen. Because of unequal experimental units/treatment after 1 entire pen of carcass data was lost, the Kenward–Rogers degrees of freedom approximation was used in the analyses of all carcass data. All carcass characteristics should be interpreted with caution. Individual carcass measurements were collected by plant personnel with the image analysis system. The proportion of cattle grading USDA Choice or greater in each pen was analyzed as a binomial proportion using the GLIMMIX procedure of SAS, with treatment as a fixed effect and block as a random effect. For all analyses, significance was determined at *P*≤0.05, with trends being defined between 0.05 <*P*≤0.10.

## RESULTS

### Growth Performance

By design, initial BW did not differ (*P* ≥ 0.60; Table 2) among treatments. There was a tendency for a 4.1% increase in final live BW (*P* = 0.06) for RES + LOC vs. LACT treatment. There were no differences in carcass-adjusted final BW (*P* ≥ 0.11) among treatments.

**Table 2.** The effects of physical activity and feed and water restriction at reimplanting time on live and carcass-adjusted growth performance of finishing beef steers

Item	Treatment^1^					SEM^2^	Contrasts^3^
	PCON	RES	LOC	RES + LOC	LACT		
*n*, steers	39	40	39	36	40	–	–
*n*, pens	10	10	10	10	10	–	–
Live weight basis^4^							
Initial body weight (**BW**), kg	385	385	385	389	388	4.9	NS
Reimplant BW, kg	509	516	511	520	506	7.7	NS
Final BW^5^, kg	626	621	619	634	608	9.5	3^†^
Average daily gain, kg							
Initial to reimplant	1.34	1.43	1.38	1.42	1.28	0.058	3^†^
Reimplant to end	1.43	1.28	1.32	1.39	1.24	0.065	3^†^,4^†^
Overall	1.41	1.34	1.36	1.39	1.32	0.029	3^†^,4^†^
Dry matter intake, kg							
Initial to reimplant	8.07	8.04	7.80	7.93	7.77	0.244	NS
Reimplant to end	8.42	8.13	7.97	8.20	7.79	0.194	5^†^
Overall	8.24	8.08	7.88	8.06	7.78	0.190	5^†^
Dry matter intake, % of BW							
Initial to reimplant	1.73	1.71	1.66	1.67	1.66	0.039	1∗,5^†^
Reimplant to end	1.43	1.37	1.35	1.36	1.34	0.023	1∗,4^†^,5^∗^
Overall	1.57	1.54	1.51	1.51	1.50	0.025	1^∗^,5^∗^
Gain:feed							
Initial to reimplant	0.167	0.178	0.177	0.180	0.166	0.0061	3^∗^
Reimplant to end	0.167	0.178	0.177	0.180	0.166	0.0061	3^∗^
Overall	0.173	0.168	0.171	0.173	0.168	0.0029	NS
Carcass-adjusted basis^6^							
Final BW, kg	617	621	618	634	608	10.2	NS
Average daily gain, kg	1.33	1.36	1.34	1.39	1.39	0.052	NS
Gain:feed	0.161	0.168	0.170	0.172	0.179	0.0053	NS

PCON = Revalor 200 [200 mg of trenbolone acetate (TBA) and 20 mg of estradiol-17β (E_2_)] given on day 90 before feeding, and steers were returned to their home pen after reimplant; RES = Revalor 200 given on day 90 before feeding, steers were then placed in sort pens and restricted from feed and water for 4 h and then returned home; LOC = Revalor 200 given on day 90 before feeding, and steers were walked an additional 805 m after reimplant and then returned home; RES + LOC = Revalor 200 given on day 90 before feeding, steers were restricted from feed and water, walked an additional 805 m, and then returned home; LACT = Revalor 200 given on day 90 with an oral bolus of *Megasphaera elsdenii* (Lactipro; MS Biotec, Wamego, KS) before feeding, steers were restricted from feed and water, and walked an additional 805 m and then returned home.

Pooled standard error of treatment means, *n* = 10 pens/treatment.

Contrasts: 1 = PCON vs. the average of RES, LOC, and RES + LOC to evaluate whether there was a difference in DMI associated with varying practices on reimplant day; 2 = RES vs. LOC to evaluate whether restriction from feed and water or additional locomotion caused a difference in DMI; 3 = RES + LOC vs. LACT to evaluate whether administering with an oral bolus of *M. elsdenii* was an effective mitigation strategy to prevent a decrease in DMI; 4 = PCON vs. RES to determine what percentage of the decrease of DMI is associated with restricting from feed and water; 5 = PCON vs. LOC to determine what percentage of the decrease of DMI is associated with locomotion.

Shrink (4%) was applied to all BW.

Blocks1 to 4 were on feed for 166 d, blocks 5 to 7 were on feed for 188 d, and blocks 8 to 10 were on feed for 166 d.

Calculated as HCW divided by overall average dressing percent (65.60%).

*P* ≤ 0.05;

0.06 ≤ *P* ≤ 0.10; NS = not significant (*P* > 0.10).

The RES + LOC treatment had a 10.8% greater ADG from reimplant to end (*P* = 0.07), and a 5% greater ADG overall (*P* = 0.10) vs. the LACT treatment. The PCON treatment tended to have a 10.5% increase in ADG from reimplant to end (*P* = 0.09) and a 5.0% increase in ADG overall (*P* = 0.08) compared to the RES treatment. There was no difference in carcass-adjusted ADG (*P* ≥ 0.12) among treatments. A tendency was detected for PCON to have a 5.3% increase in DMI (*P* = 0.10) from reimplant to end and a 4.4% increase in overall DMI (*P* = 0.10) compared to the LOC treatment.

The PCON treatment tended to have a 4.2% greater DMI (*P *= 0.10) as a percentage of BW than the RES treatment from reimplant to end. As a percentage of BW, DMI was 5.6% greater (*P* = 0.03) from reimplant to end and 3.8% greater (*P *= 0.05) overall for the PCON treatment than the LOC treatment. Overall, DMI as a percentage of BW was 3.2% greater (*P* = 0.02) for PCON vs. RES, LOC, and RES + LOC treatments.

**Figure 1.**
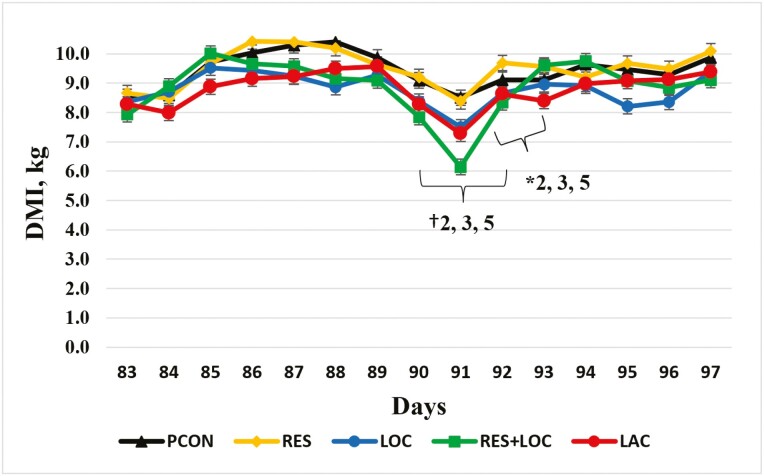
Dry matter intake 7 d before and after reimplanting for finishing steers. Reimplant was given on day 90. PCON = Revalor 200 [200 mg of trenbolone acetate (TBA) and 20 mg of estradiol-17β (E_2_)] given on day 90 before feeding, and steers were returned to their home pen after reimplant; RES = Revalor 200 given on day 90 before feeding, steers were then placed in sort pens and restricted from feed and water for 4 h and then returned to their home pen; LOC = Revalor 200 given on day 90 before feeding, and steers were walked an additional 805 m after reimplant and then returned to their home pen; RES + LOC = Revalor 200 given on day 90 before feeding, steers were restricted from feed and water, walked an additional 805 m, and then returned to their home pen; LACT = Revalor 200 given on day 90 with an oral bolus of *Megasphaera elsdenii* (Lactipro; MS Biotec, Wamego, KS) before feeding, steers were restricted from feed and water, walked an additional 805 m, and then returned to their home pen. Treatment *P* = 0.09, Day *P* < 0.01, Treatment × Day *P* = 0.05. Within day, ∗*P* < 0.05 for contrasts specified; † 0.06 < *P* < 0.10 for contrasts specified.

**Figure 2.**
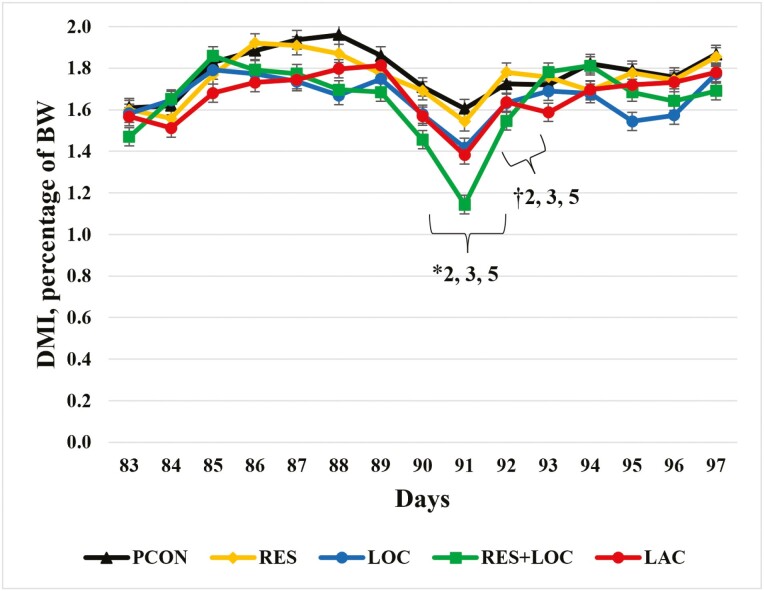
Dry matter intake as a percentage of BW 7 d before and after reimplanting for finishing steers. Reimplant was given on day 90. PCON = Revalor 200 [200 mg of trenbolone acetate (TBA) and 20 mg of estradiol-17β (E_2_)] given on day 90 before feeding, and steers were returned to their home pen after reimplant; RES = Revalor 200 given on day 90 before feeding, steers were then placed in sort pens and restricted from feed and water for 4 h and then returned to their home pen; LOC = Revalor 200 given on day 90 before feeding, and steers were walked an additional 805 m after reimplant and then returned to their home pen; RES + LOC = Revalor 200 given on day 90 before feeding, steers were restricted from feed and water, walked an additional 805 m, and then returned to their home pen; LACT = Revalor 200 given on day 90 with an oral bolus of *Megasphaera elsdenii* (Lactipro; MS Biotec, Wamego, KS) before feeding, steers were restricted from feed and water, walked an additional 805 m, and then returned to their home pen. Treatment *P* = 0.10, Day *P* < 0.01, Treatment × Day *P* = 0.04. Within day, ∗*P* < 0.05 for contrasts specified; † 0.06 < *P* < 0.10 for contrasts specified.

Dry matter intake 7 d before and after reimplanting is presented in Figures 1 and 2 where reimplant was given on day 90. There was a tendency for DMI to be 6.9% greater (*P *= 0.07) for the RES treatment on day 90 and 8.4% greater (*P *= 0.05) on day 91 than the LOC treatment as a percentage of BW. The LACT treatment tended to have a 7.5% increase (*P* = 0.09) in DMI on day 90 and a 21% increase on day 91 (*P* = 0.04) compared to the RES + LOC treatment as a percentage of BW. On day 90, as a percentage of BW, the PCON treatment tended to have an 8.2% increase (*P* = 0.07) in DMI than the LOC treatment. Likewise, on day 91, as a percentage of BW, the PCON treatment tended to have a 13.3% increase (*P* = 0.04) in DMI than the LOC treatment.

A 7.8% increase in G:F (*P *= 0.05; Table 2) from reimplant to end (*P *= 0.05) was noted for RES + LOC compared to the LACT treatment. There were no differences in carcass-adjusted G:F (*P* ≥ 0.11) among treatments.

### Activity Data

**Figure 3.**
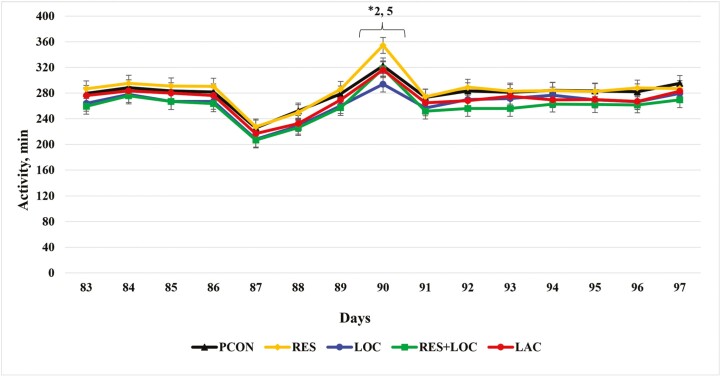
Daily activity (minutes) 7 d before and after reimplanting for finishing steers. Reimplant was given on day 90. PCON = Revalor 200 [200 mg of trenbolone acetate (TBA) and 20 mg of estradiol-17β (E_2_)] given on day 90 before feeding, and steers were returned to their home pen after reimplant; RES = Revalor 200 given on day 90 before feeding, steers were then placed in sort pens and restricted from feed and water for 4 h, and then returned to their home pen; LOC = Revalor 200 given on day 90 before feeding, and steers were walked an additional 805 m after reimplant and then returned to their home pen; RES + LOC = Revalor 200 given on day 90 before feeding, steers were restricted from feed and water and walked an additional 805 m and then returned to their home pen; LACT = Revalor 200 given on day 90 with an oral bolus of *Megasphaera elsdenii* (Lactipro; MS Biotec, Wamego, KS) before feeding, steers were restricted from feed and water, walked an additional 805 m, and then returned to their home pen. Treatment *P* = 0.15, Day *P* < 0.01, Treatment × Day *P* = 0.05. Within day, ∗*P* < 0.05 for contrasts specified; † 0.06 < *P* < 0.10 for contrasts specified.

There was a treatment × day interaction (*P* < 0.01; Figure 3) for daily minutes of activity 7 d before and after reimplanting. On day 90 when the steers were reimplanted, the PCON treatment was 9.6% more active (*P* = 0.05) than the LOC treatment. The RES treatment had 20% more minutes of activity (*P* = 0.03) than the LOC treatment on day 90.

### Carcass Characteristics

Carcass data are presented in Table 3. The RES + LOC treatment tended to have a 3.8% greater (*P* = 0.09) HCW than the LACT treatment. There were no differences among treatments in dressing percent, longissimus area, 12th rib fat, or kidney, pelvic, heart fat (KPH; *P* ≥ 0.13). There was a tendency for the LOC treatment to have a 7.7% increase in marbling (*P* < 0.08) than the RES treatment. The RES + LOC treatment tended to have a 6.9% increase in marbling (*P* < 0.09) compared to the LACT treatment. The PCON treatment had a 9.2% increase in marbling (*P* < 0.02) compared to the RES treatment. There were no differences in Quality Grade or Yield Grade among treatments (*P* ≥ 0.12). The RES, LOC, and RES + LOC treatments tended to have a 4.8% increase in adjusted final BW (AFBW; *P* < 0.08) compared to the PCON treatment.

**Table 3.** The effects of physical activity and feed and water restriction at reimplanting time on carcass characteristics of finishing beef steers

Item	Treatment^1^					SEM^2^	Contrasts^3^
	PCON	RES	LOC	RES + LOC	LACT		
Hot carcass weight, kg	405	408	404	419	403	6.0	3^†^
Dressing percent^4^, %	64.69	65.70	65.27	66.08	66.28	0.393	NS
LM^4^ area, cm sq	87.85	89.50	88.34	91.85	89.11	2.708	NS
12th-rib fat, cm	1.84	1.64	1.69	1.61	1.57	0.125	NS
KPH, %	5.01	4.95	4.74	4.99	4.70	0.196	NS
Yield Grade	3.43	3.19	3.27	3.15	3.09	0.216	NS
Marbling score^5^	544	494	532	507	545	17.4	1^†^,2^†^,3^†^,4^∗^
Choice or greater, %	95.00	86.67	89.17	94.84	95.00	4.783	NS
EBF^6^, %	32.21	31.28	31.53	31.34	30.92	0.650	NS
AFBW^7^, kg	543	568	557	583	568	13.7	1^†^

PCON = Revalor 200 [200 mg of trenbolone acetate (TBA) and 20 mg of estradiol-17β (E_2_)] given on day 90 before feeding, and steers were returned to their home pen after reimplant; RES = Revalor 200 given on day 90 before feeding, steers were then placed in sort pens and restricted from feed and water for 4 h and then returned to home; LOC = Revalor 200 given on day 90 before feeding, and steers were walked an additional 805 m after reimplant and then returned home; RES + LOC = Revalor 200 given on day 90 before feeding, steers were restricted from feed and water, walked an additional 805 m, and then returned home; LACT = Revalor 200 given on day 90 with an oral bolus of *Megasphaera elsdenii* (Lactipro; MS Biotec, Wamego, KS) before feeding, steers were restricted from feed and water, walked an additional 805 m, and then returned home.

Pooled standard error of treatment means, *n* = 10 pens/treatment.

Contrasts: 1 = PCON vs. the average of RES, LOC, and RES + LOC to evaluate whether there was a difference in DMI associated with varying practices on reimplant day; 2 = RES vs. LOC to evaluate whether restriction from feed and water or additional locomotion caused a difference in DMI; 3 = RES + LOC vs. LACT to evaluate whether administering with an oral bolus of *M. elsdenii*was an effective mitigation strategy to prevent a decrease in DMI; 4 = PCON vs. RES to determine what percentage of the decrease of DMI is associated with restricting from feed and water; 5 = PCON vs. LOC to determine what percentage of the decrease of DMI is associated with locomotion.

Calculated as HCW divided by final shrunk BW.

Leading digit in marbling indicates score; 2 = trace, 3 = slight, 4 = small, 5 = modest, 6 = moderate, 7 = slightly abundant, 8 = moderately abundant, 9 = abundant. Following digits indicate degree of marbling within marbling score.

Empty body fat was calculated using equations of Guiroy et al. (2001).

Adjusted final shrunk BW at 28% empty body fat was estimated using equations of Tylutki et al. (1994).

*P* ≤ 0.05;

0.06 ≤ *P* ≤ 0.10; NS = not significant (*P* > 0.10).

## DISCUSSION

The effects of TBA and E_2_ combination implants in beef cattle have been extensively studied since their approval in 1991 (Preston, 1999). Implanting and reimplanting cattle allows for a hormone payout throughout the entire feeding period, but reimplanting must be done in a way to limit stress on cattle (Stanton, 1997; Smith et al., 2018). A decrease in DMI caused by stress can lead to decreased growth performance and carcass characteristics with a subsequent increase in cost of gain (Stanton, 1997).

The tendency for a difference in final BW for RES + LOC vs. LACT was caused by increased ADG from reimplant to end and overall, though the increased ADG was not caused by increased DMI or DMI as a percentage of BW. The PCON treatment had a greater ADG from reimplant to end and overall compared to the RES treatment. The increase in ADG is likely because the PCON treatment had a greater DMI as a percentage of BW than the RES treatment from reimplant to end.

In the present study, there was a tendency for PCON to have a greater DMI overall when compared with the RES, LOC, RES + LOC, and all steers received the same reimplant. As a percentage of BW, cattle that were on the RES, LOC, and RES + LOC treatments had a 3.3% decrease in DMI. Taken together, these data suggest that management practices such as restricting cattle from feed and water during the reimplant process and additional locomotion on reimplant day contributed to a decrease in DMI.

Although no statistical differences were noted, DMI decreased for 1 day following reimplanting (day 91 on Figures 1 and 2) among all treatments. The steers on the LOC, RES + LOC, and LACT treatment had decreased DMI and DMI as a percentage of BW than the other treatments the day after reimplanting. This indicates that the combination of feed and water restriction with locomotion or locomotion alone contributed to a sharp decrease in DMI after reimplanting. Interestingly, the bolus of *M. elsdenii* used in the LACT treatment mitigated some decrease in DMI (kg and as percentage of BW) on day 91, as receiving the *M. elsdenii* bolus was the only management difference between the RES + LOC and LACT treatments. Although the decrease in DMI was pronounced 1 d after reimplanting (day 91), DMI increased quickly in all treatments during the days following reimplanting. Similarly, Wallace et al. (2008) reported that on average cattle decrease DMI for 3 to 4 d after reimplanting. In the present experiment, DMI decreased for 1 d after reimplant before starting to increase. Wallace et al. (2008) noted that cattle consumed less feed for 10 d after reimplanting if using the 10 d before reimplanting as the baseline. Differences in the present experiment and the results of Wallace et al. (2008) could be attributed to pen size, commercial vs. research setting, or cattle handling techniques.

Difference in activity, which includes eating, rumination, and locomotion at different speeds, can be related to the variation in DMI in cattle (Herd and Arthur, 2009). Herd et al. (2004) and Richardson et al. (1999) concluded that 5% to 10 % of the variation in DMI could be associated with activity. In the present study, increased locomotion on reimplant day was associated with a 5.6% reduction in DMI from reimplant to end and a 4.6% decrease over the entire feeding period. Nonetheless, activity measured from the 3-axis accelerometer tags includes time spent eating; thus, the activity measurement may be slightly confounded where the treatments that consumed more DMI would also have the greatest activity. Typically, cattle consuming high-concentrate diets spend between 10 and 69 min/d (Islam et al., 2021) and 8 to 89 min/d (Gibb et al., 2010) consuming feed. Davis et al. (2014) monitored time eating (min/d) in 143 steers fed a high-concentrate diet with slightly lower energy concentration than the one used in the current study. Over the 106-d period, cattle spent 133 min/day eating. Physical activity can affect total energy expenditure and if the energy expended is not compensated by an increase in DMI overall gain and efficiency can be decreased (Susenbeth et al., 1998; Herd et al., 2004; Herd and Arthur, 2009). Llonch et al. (2018) reported greater DMI for cattle that took fewer steps. There was a dramatic decrease in activity 3 d before reimplanting that can be attributed to abnormally cold weather 7 d before reimplanting in blocks 5, 6, and 7 (February 17 to February 24, 2021; high temperature −8 °C, low temperature −18 °C; The National Weather Service).

Cattle that were restricted from feed and water for 4 h on reimplant day (RES treatment) were the most active on reimplant day which was unexpected. Although not measured, we speculate that the increase in activity for the RES treatment may be the result of the cattle being unsettled when housed in an unfamiliar pen causing additional locomotion within the holding pen, thereby increasing their minutes of activity. Although the LOC treatment was walked 805 m on day of reimplant, they were less active compared to the PCON and RES treatment. This was an unexpected finding and is likely because of exhaustion from being forced to walk 805 m causing the steers to be less active when returned home to their pen. We hypothesized that *M. elsdenii* would be an effective strategy to mitigate the decrease in DMI following reimplant. Although not significant, the LACT treatment had slightly greater DMI 7 d after reimplanting compared to the RES + LOC treatment. These results indicate that *M. elsdenii* may help mitigate an acute decrease in DMI associated with reimplant, but it was not effective across the entire feeding period. The RES + LOC treatment had a greater HCW compared to the LACT treatment; however, because of missing data especially in the RES + LOC treatment (9 steers), this difference should be interpreted with caution.

Implanting and reimplanting is a common practice in the cattle feeding industry which has been associated with depressed feed intake in subsequent days. Cattle management before, during, and after implanting can markedly influence DMI. From the current study, we conclude that returning cattle to their home pen immediately after reimplanting is an effective method to mitigate a decrease in DMI. Restricting cattle from feed and water for 4 h after leaving their home pen did not change DMI following reimplant. These results indicate if cattle must be staged in holding pens without access to feed and water for up to 4 h during the reimplanting process, risk of detriment to DMI is low compared to increased locomotion associated with reimplanting. Locomotion on reimplant day had the greatest negative effect on DMI and subsequent growth performance. Management strategies to decrease locomotion associated with reimplanting would be beneficial to DMI and overall growth performance of finishing beef steers.

## References

[CIT0001] APHIS (Animal and Plant Health Inspection Services). 2013. The use of growth-promoting implants in U.S. feedlots. USDA.

[CIT0002] Bartle, S. J., R. L. Preston, R. E. Brown, and R. J. Grant. 1992. Trenbolone acetate/estradiol combinations in feedlot steers: dose-response and implant carrier effects. J. Anim. Sci. 70:1326–1332. doi:10.2527/1992.7051326x. 1526900

[CIT0003] Davis, M. P., H. C. Freetly, L. A. Kuehn, J. E. Wells. 2014. Influence of dry matter intake, dry matter digestibility, and feeding behavior on body weight gain of beef steers. J. Anim. Sci. 92:3018–3025. doi:10.2527/jas.2013-6518. 24802034

[CIT0004] Gibb, D. J., T. A. McAllister, C. Huisma, and R. D. Wiedmeier. 2010. Bunk attendance of feedlot cattle monitored with radio frequency technology. Can. J. Anim. Sci. 78:707–710. doi:10.1139/cjas-2015-0193.

[CIT0005] Guiroy, P. J., D. G. Fox, L. O. Tedeschi, M. J. Baker, and M. D. Cravey. 2001. Predicting individual feed requirements of cattle fed in groups. J. Anim. Sci. 79:1983–1995. doi:10.2527/2001.7981983x. 11518207

[CIT0006] Henning, P. H., C. H. Horn, K. J. Leeuw, H. H. Meissner, and F. M. Hagg. 2010. Effect of ruminal administration of the lactate-utilizing strain *Megasphaera elsdenii* (Me) NCIMB 41125 on abrupt or gradual transition from forage to concentrate diets. Anim. Feed Sci. Tech. 157:20–29. doi:10.1016/j.anifeedsci.2010.02.00.2.

[CIT0007] Herd, R. M., and P. F. Arthur. 2009. Physiological basis for residual feed intake. J. Anim. Sci. 87:E64–E71. doi:10.2527/jas.2008-1345. 19028857

[CIT0008] Herd, R. M., V. H. Oddy, and E. C. Richardson. 2004. Biological basis for variation in residual feed intake in beef cattle. 1. Review of potential mechanisms. Aust. J. Exp. Agric. 44:423–430. doi: 10.1071/EA02221.

[CIT0009] Islam, M. A., S. Lomax, A. K. Doughty, M. R. Islam, and C. E. F. Clark. 2021. Timing of eating during transition impacts feedlot cattle diet and liveweight gain. Animal 3:100137. doi:10.1016/j.animal2020:100137. 33573939

[CIT0010] Llonch, P., M. Somarriba, C. A. Duthie, S. Troy, R. Roehe, J. Rooke, M. J. Haskell, and S. Turner. 2018. Temperament and dominance relate to feeding behaviour and activity in beef cattle: implications for performance and methane emissions. Animal 12:2639–2648. doi:10.1017/S175173111800061.7. 29606168

[CIT0011] Mobiglia, A. M., F. R. Camilo, and J. S. Drouillard. 2016. 1630 substrate utilization by *Megasphaera elsdenii* strain NCIMB 41125. J. Anim. Sci. 94(Suppl_5):793–794. doi: 10.2527/jam2016-162.9.

[CIT0012] NASEM. 2016. The National Academics of Sciences Engineering and Medicine Nutrient requirements of beef cattle. 8th rev. ed. National Academies Press, Washington, DC.

[CIT0013] Owens, F. N., D. S. Secrist, W. J. Hill, and D. R. Gill. 1998. Acidosis in cattle: a review. J. Anim. Sci. 76:275–286. doi:10.2527/1998.761275x. 9464909

[CIT0014] Preston, R. L. 1999. Hormone containing growth promoting implants in farmed livestock. Adv. Drug Deliv. Rev. 38:123–138. doi:10.1016/S0169-409X(99)00012-5 10837752

[CIT0015] Reinhardt, C. D., and J. J. Wagner. 2014. High-dose anabolic implants are not all the same for growth and carcass traits of feedlot steers: a meta-analysis. J. Anim. Sci. 92:4711–4718. doi:10.2527/jas.2014-7572. 25149344

[CIT0016] Richardson, E. C., R. J. Kilgour, J. A. Archer, and R. M. Herd. 1999. Pedometers measure differences in activity in bulls selected for high or low net feed efficiency. Aust. Soc. Anim. Behav. 26:16–2.

[CIT0017] Smith, Z. K., A. J. Thompson, J. P. Hutcheson, W. T. Nichols, and B. J. Johnson. 2018. Evaluation of coated steroidal implants containing trenbolone acetate and estradiol-17β on live performance, carcass traits, and sera metabolites in finishing steers. J. Anim. Sci. 96:1704–1723. doi:10.1093/jas/sky095. 29534183 PMC6140838

[CIT0018] Stanton, T. 1997. Cost of reworking cattle. P-95 7:95-99. Oklahoma Agricultural Experiment Station, Oklahoma State University, Stillwater, OK.

[CIT0019] Susenbeth, A., R. Mayer, B. Koehler, and O. Neumann. 1998. Energy requirement for eating in cattle. J. Anim. Sci. 76:2701–2705. doi:10.2527/1998.76102701x. 9814912

[CIT0020] Tylutki, T. P., D. G. Fox, and R. G. Anrique. 1994. Predicting net energy and protein requirements for growth of implanted and nonimplanted heifers and steers and nonimplanted bulls varying in body size. J. Anim. Sci. 72:1806–1813. doi:10.2527/1994.7271806x. 7928760

[CIT0021] Wallace, J. O., C. D. Reinhardt, W. T. Nichols, J. P. Hutcheson, B. J. Johnson, and J. S. Drouillard. 2008. The costs associated with reimplanting. Plains Nutr. Counsil. Spring Conf., San Antonio, TX, AREC 08-19. Texas AgriLife Res. Ext. Center, Texas A&M, Amarillo.

[CIT0022] Wolfger, B., E. Timsit, E. A. Pajor, N. Cook, H. W. Barkema K. Orsel. 2015. Technical note: accuracy of an ear tag-attached accelerometer to monitor rumination and feeding behavior in feedlot cattle. J. Anim. Sci. 93:3164–3168. doi:10.2527/jas2014-8802. 26115302

